# Identifying tumor immunity-associated molecular features in liver hepatocellular carcinoma by multi-omics analysis

**DOI:** 10.3389/fmolb.2022.960457

**Published:** 2022-10-20

**Authors:** Qianyun Shen, Yin He, Jiajie Qian, Xiaosheng Wang

**Affiliations:** ^1^ Department of Gastrointestinal Surgery, The First Affiliated Hospital, College of Medicine, Zhejiang University, Hangzhou, China; ^2^ Biomedical Informatics Research Lab, School of Basic Medicine and Clinical Pharmacy, China Pharmaceutical University, Nanjing, China; ^3^ Cancer Genomics Research Center, School of Basic Medicine and Clinical Pharmacy, China Pharmaceutical University, Nanjing, China; ^4^ Big Data Research Institute, China Pharmaceutical University, Nanjing, China

**Keywords:** hepatocellular carcinoma, antitumor immunity, cancer immunotherapy, multi-omics analysis, biomarker

## Abstract

**Background:** Although current immunotherapies have achieved some successes for hepatocellular carcinoma (HCC) patients, their benefits are limited for most HCC patients. Therefore, the identification of biomarkers for promoting immunotherapeutic responses in HCC is urgently needed.

**Methods:** Using the TCGA HCC cohort, we investigated correlations of various molecular features with antitumor immune signatures (CD8^+^ T cell infiltration and cytolytic activity) and an immunosuppressive signature (PD-L1 expression) in HCC. These molecular features included mRNAs, microRNAs (miRNAs), long non-coding RNAs (lncRNAs), proteins, and pathways.

**Results:** We found that the mutations of several oncogenes and tumor suppressor genes significantly correlated with reduced antitumor immune signatures, including *TTN*, *CTNNB1*, *RB1*, *ZFHX4*, and *TP53*. It indicates that these genes’ mutations may inhibit antitumor immune responses in HCC. Four proteins (Syk, Lck, STAT5, and Caspase-7) had significant positive expression correlations with CD8^+^ T cell enrichment, cytolytic activity, and PD-L1 expression in HCC. It suggests that these proteins’ expression could be useful biomarkers for the response to immune checkpoint inhibitors Similiarly, we identified other types of biomarkers potentially useful for predicting the response to ICIs, including miRNAs (hsa-miR-511-5p, 150-3p, 342-3p, 181a-3p, 625-5p, 4772-3p, 155-3p, 142-5p, 142-3p, 155-5p, 625-3p, 1976, 7702), many lncRNAs, and pathways (apoptosis, cytokine-cytokine receptor interaction, Jak-STAT signaling, MAPK signaling, PI3K-AKT signaling, HIF-1 signaling, ECM receptor interaction, focal adhesion, and estrogen signaling). Further, tumor mutation burden showed no significant correlation with antitumor immunity, while tumor aneuploidy levels showed a significant negative correlation with antitumor immunity.

**Conclusion:** The molecular features significantly associated with HCC immunity could be predictive biomarkers for immunotherapeutic responses in HCC patients. They could also be potential intervention targets for boosting antitumor immunity and immunotherapeutic responses in HCC.

## Introduction

Hepatocellular carcinoma (HCC) is a primary malignancy of the liver and accounts for around 90% of cases (Villanueva, 2019). Traditional therapeutic strategies, such as surgical interventions, radiotherapy, transarterial therapies, chemotherapy, and targeted therapy, are common approaches to HCC treatment. Although these therapies have substantially improved the survival of HCC patients, median survival times for early, intermediate, and advanced HCC are around 36, 16, and 6 months, respectively (Llovet et al., 2021). Recently, immunotherapies have demonstrated successes in treating various malignancies, including HCC (Yau et al., 2020). In 2020, the combination of atezolizumab (anti-PD-L1 antibody) and bevacizumab (anti-VEGF antibody) was approved as first-line therapy for HCC. It indicates clinical benefit of immunotherapies for HCC patients. Nevertheless, only a subset of cancer patients currently benefit from immunotherapies (Braun et al., 2016). The mechanisms of response and resistance to immunotherapies remains unclear, and potential predictive biomarkers remain to be uncovered. To this end, certain genetic or genomic markers for cancer immunotherapeutic responses have been identified, such as PD-L1 expression (Taube et al., 2014), tumor mutation burden (TMB) (Goodman et al., 2017), and mismatch repair deficiency (Le et al., 2015). Besides, the tumor immune microenvironment (TIME) is a key regulator of immunotherapeutic responses (Murciano-Goroff et al., 2020). In general, the “hot” tumors infiltrated by ample tumor-infiltrating lymphocytes (TILs) respond better to immunotherapies, compared to the “cold” tumors with sparse TILs (Haanen, 2017).

Based on large-scale cancer genomics data, such as TCGA (https://portal.gdc.cancer.gov/) and ICGC (https://dcc.icgc.org/), many studies have explored the TIME in HCC (Sia et al., 2017; Farha et al., 2020; Gao et al., 2020). For example, Gao et al. identified four immune-related subtypes of HCC based on the abundances of 13 tumor microenvironment (TME)-associated signatures, which displayed significantly different molecular and clinical characteristics (Gao et al., 2020). Sia et al. identified an immune-specific subclass of HCC by analysis of gene expression profiles in tumor, stromal, and immune cells, which constituted approximately 25% of HCC cases (Sia et al., 2017). Farha et al. identified two immune clusters of HCC based on the enrichment of immune cell subpopulations, of which the M0 macrophage-enriched cluster had a poor prognosis (Farha et al., 2020).

Despite these prior investigations (Sia et al., 2017; Farha et al., 2020; Gao et al., 2020), a comprehensive identification of molecular features significantly associated with the TIME in HCC is worth further investigation, because these molecular features could be predictive biomarkers for the response to immunotherapy in HCC. In this study, by multi-omics analysis, we explored the associations of various molecule types with HCC immunity in the TCGA HCC cohort. These molecule types included genes (DNA and mRNA), microRNAs (miRNAs), long non-coding RNAs (lncRNAs), proteins, and pathways. We identified the molecular features showing significant correlations with antitumor immune signatures (such as CD8^+^ T cell infiltration and cytolytic activity) and immunosuppressive signatures (such as PD-L1 expression) in HCC. This study aimed to discover novel predictive biomarkers for the immunotherapeutic response in HCC that have potential clinical values.

## Methods

### Dataset

We downloaded multi-omics (somatic mutations, mRNA expression, protein expression, miRNA expression, and lncRNA expression) and clinical data for the TCGA HCC cohort from the GDC data portal (https://portal.gdc.cancer.gov/). All expression values (RSEM normalized) were transformed by log2 (x+1) before subsequent analyses, except protein expression values which were normalized in TCGA generated by the reverse phase protein array (RPPA). In addition, we obtained cancer-associated pathways and their gene sets from KEGG (Kanehisa et al., 2017).

### Quantification of immune signature enrichment in tumors

The enrichment level of an immune signature in a tumor was defined as the mean expression level of its marker genes. A total of three immune signatures were analyzed, including CD8^+^ T cells, cytolytic activity, and PD-L1 expression. The marker genes of these immune signatures are shown in Table 1.

**TABLE 1:** The marker genes of the three immune signatures analyzed in this study.

Immune signature	Marker genes
CD8^+^ T cells	*CD2*, *CD247*, *CD28*, *CD3D*, *CD3E*, *CD3G*, *CD8A*, *ICAM1*, *ITGAL*, *ITGB2*, *PTPRC*, *THY1*
Cytolytic activity	*PRF1*, *GZMA*
PD-L1	*PD-L1*

### Correlations between immune signature enrichment and molecular features in hepatocellular carcinoma

We identified the genes whose mutations were significantly correlated with immune signature enrichment in HCC using Student’s *t* tests. Pearson correlation tests were utilized to identify the genes, proteins, miRNAs, and lncRNAs having significant expression correlations with immune signature enrichment in HCC. We quantified a pathway’s activity in a tumor by the single-sample gene-set enrichment analysis (ssGSEA) (Hanzelmann et al., 2013). The ssGSEA algorithm evaluated a pathway’s activity, namely ssGSEA score, in a sample based on the expression profiles of the pathway gene set. The R package “GSVA” was utilized to perform the ssGSEA algorithm. Spearman correlation tests were used to identify the cancer-associated pathways whose activity significantly correlated with immune signature enrichment in HCC. The false discovery rate (FDR) (Benjamini and Hochberg, 1995) was used to correct for *p*-values in multiple tests.

### Gene-set enrichment analysis

We used GSEA (Subramanian et al., 2005) to identify the KEGG (Kanehisa et al., 2017) pathways that were significantly associated with the genes having an important expression correlation with immune signature enrichment with a threshold of FDR <0.05. Given an *a priori* defined set of genes *S*, GSEA will predict pathways, biological function, and other phenotypes significantly associated with *S*, according to whether the members of S are randomly distributed throughout the entire ranked list *L* or primarily found at the top or bottom.

### Quantification of TMB and tumor aneuploidy level (TAL)

We defined a tumor’s TMB as the total somatic mutation count in the tumor. A tumor’s TAL was its tumor ploidy score calculated by absolute (Carter et al., 2012), which estimates the tumor ploidy score based on its somatic copy number alterations with the input of segmented copy number data.

### Survival analysis

We compared the overall survival rate between gene-mutated and gene-wildtype cancers in the TCGA HCC cohort. We used Kaplan-Meier survival curves to show the survival time differences and the log-rank test to assess the significance of survival time differences.

## Results

### Identification of genes whose mutations correlate with antitumor immune responses in hepatocellular carcinoma

**FIGURE 1:**
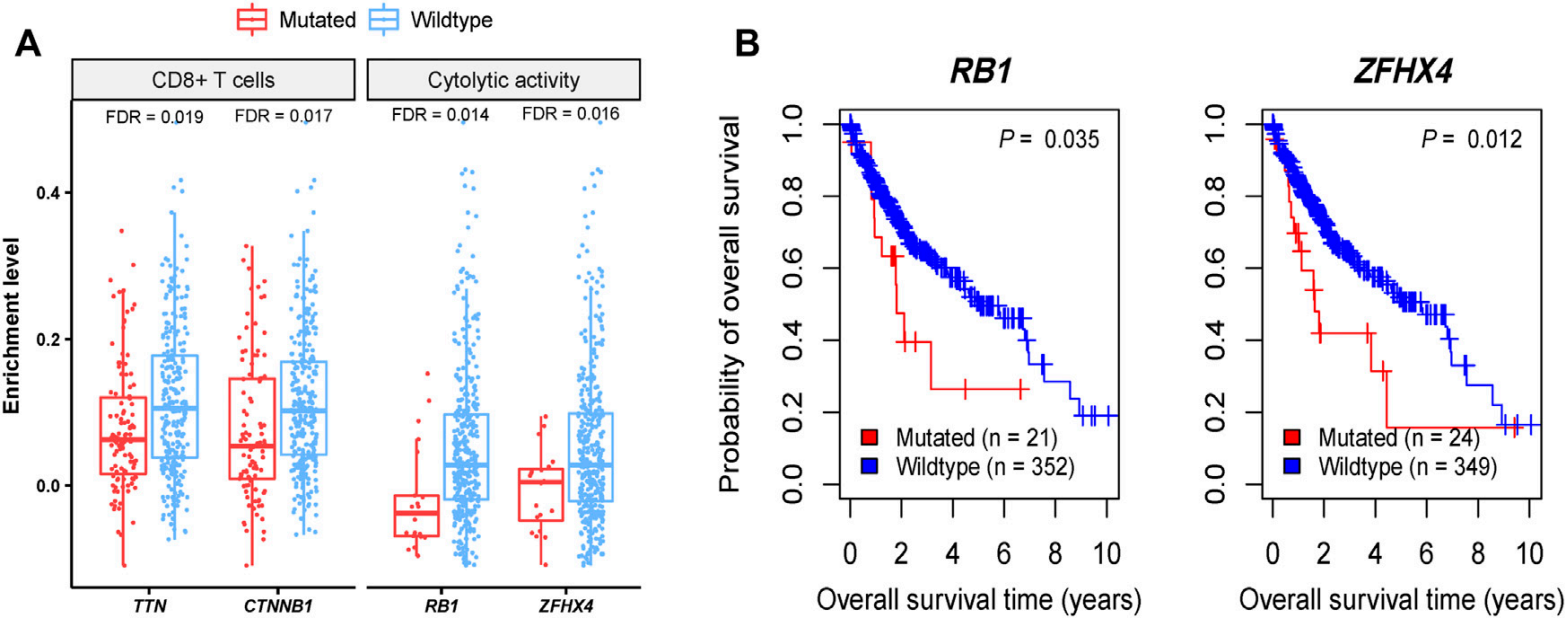
Four genes whose mutations have significant correlations with reduced antitumor immune responses in HCC. **(A)** Four genes whose mutations are correlated with reduced CD8^+^ T cell enrichment levels or cytolytic activity in HCC. **(B)** Kaplan-Meier curves showing two of the four genes whose mutations are correlated with better overall survival in HCC.

We found four genes whose mutations showed significant correlations with reduced CD8^+^ T cell enrichment levels or cytolytic activity in HCC (Mann-Whitney *U* test, FDR <0.05), including *TTN*, *CTNNB1*, *RB1*, and *ZFHX4* (Figure 1A). Also, *TP53* mutations were associated with reduced cytolytic activity in HCC (Mann-Whitney *U* test, *p* = 0.001). These results indicate that these genes’ mutations may inhibit antitumor immune responses in HCC. *CTNNB1* encodes a protein that functions on regulating cell growth and adhesion between cells and is a regulator of the WNT signaling pathway (He and Tang, 2020). *CTNNB1* (catenin beta 1) mutations are involved in development of various cancers, including colorectal cancer (Akyol et al., 2019), lung cancer (Zhou et al., 2021), HCC (Kalasekar et al., 2019), ovarian and endometrial endometrioid cancer (Liu et al., 2014), Wilms tumors (Li et al., 2004), and medulloblastoma (Fattet et al., 2009). The Wnt/β-catenin pathway is involved in modulating antitumor immune responses (Pai et al., 2017), supporting our findings. *TP53* (tumor protein p53) is a well-known tumor suppressor gene and is mutated in a wide variety of human cancers (Wang and Sun, 2017). This gene is the second most frequently mutated genes, next to *TTN*, with a mutation rate of more than 30% in HCC (Sun et al., 2018). The association between *TP53* mutations and decreased antitumor immune responses has been uncovered in many cancers, such as head and neck squamous cell cancer (Lyu et al., 2019), gastric cancer (Jiang et al., 2018), and colon cancer (Li et al., 2020a). It is consistent with the finding in this study. *ZFHX4* (zinc finger homeobox 4) has a mutation rate of around 6% in HCC, and its mutations are associated with unfavorable overall survival in HCC (Sun et al., 2018). Although this gene is mutated in various cancer types, such as lung cancer, melanoma, gastrointestinal cancer, and bladder cancer (Sun et al., 2018), few studies have reported the association between its mutations and antitumor immunity. *RB1* (RB transcriptional corepressor 1) is another tumor suppressor gene, with a mutation rate of nearly 6% in HCC (Sun et al., 2018). A previous study has shown that co-mutations of *RB1* and *TP53* were associated with increased antitumor immune responses in bladder cancer (Manzano et al., 2021), contrast with the findings in this study. It indicates the cancer type-dependent association between their mutations and antitumor immune responses, as reported in a previous study (Li et al., 2020a). Among the four genes, mutations of *RB1* and *ZFHX4* were associated with a lower overall survival rate in HCC (Figure 1B). It is justified since their mutations are associated with reduced antitumor immune responses.

### Identification of genes whose expression correlates with antitumor immune responses in hepatocellular carcinoma

**FIGURE 2:**
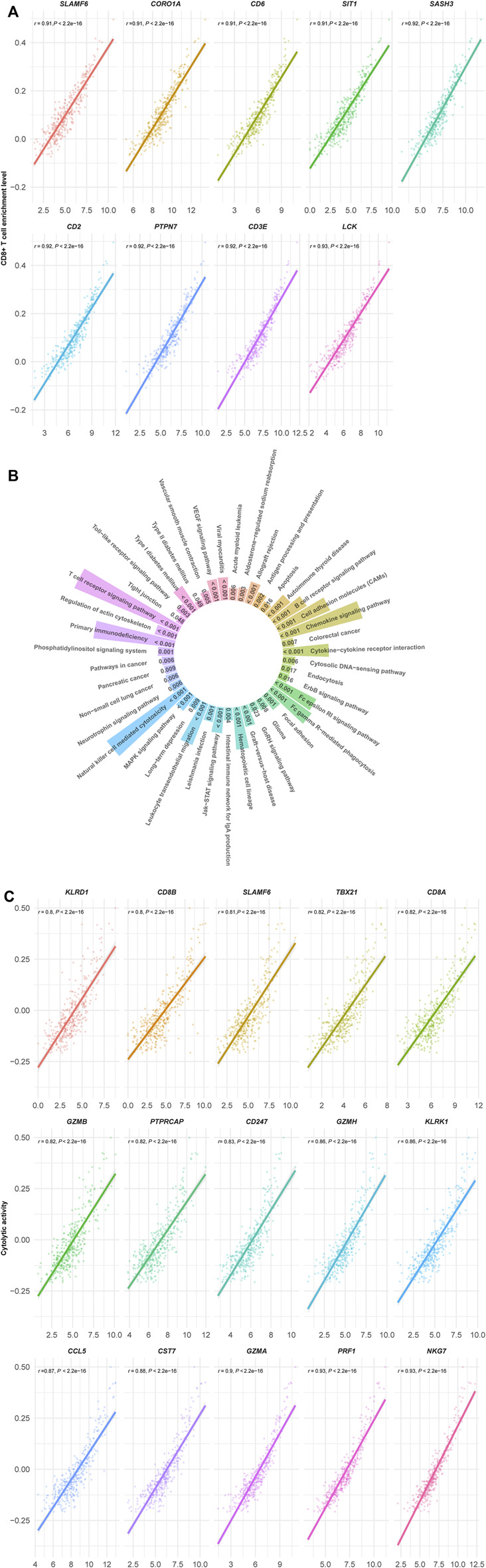
(Continued).

We found 140 genes having strong positive expression correlations with the enrichment levels of CD8^+^ T cells in HCC (Pearson correlation coefficient *r* > 0.8) (Supplementary Table S1). Among them, nine genes showed the strongest positive expression correlations with the enrichment levels of CD8^+^ T cells in HCC (*r* > 0.9), including *SLAMF6*, *COR O 1A*, *CD6*, *SIT1*, *SASH3*, *CD2*, *PTPN7*, *CD3E*, and *LCK* (Figure 2A). As expected, all these genes are involved in the regulation of T cell activation (Chetty and Gatter, 1994; Marie-Cardine et al., 1999; Beer et al., 2001; Palacios and Weiss, 2004; Mueller et al., 2008; Stanford et al., 2012; Santos et al., 2016; Dragovich et al., 2019; Zurli et al., 2020). GSEA (Subramanian et al., 2005) identified 43 KEGG (Kanehisa et al., 2017) pathways significantly correlated with the 140 genes (Figure 2B). As expected, most of these pathways are involved in immune regulation, such as T cell receptor signaling, natural killer cell-mediated cytotoxicity, chemokine signaling, cytokine-cytokine receptor interaction, cell adhesion molecules, Jak-STAT signaling, antigen processing and presentation, cytosolic DNA-sensing, and Toll-like receptor signaling. In addition, the 43 pathways included certain stromal and oncogenic pathways, including regulation of actin cytoskeleton, focal adhesion, tight junction, VEGF, MAPK, and ErbB signaling pathways. Besides, we found 15 genes showing strong positive expression correlations with cytolytic activity in HCC (*r* > 0.8) (Figure 2C). These genes included *KLRD1*, *CD8B*, *SLAMF6*, *TBX21*, *CD8A*, *GZMB*, *PTPRCAP*, *CD247*, *GZMH*, *KLRK1*, *CCL5*, *CST7*, *GZMA*, *PRF1*, and *NKG7*. Again, all these genes are involved in immune regulation. Furthermore, we did not found genes whose expression had strong positive correlations with *PD-L1* expression (*r* < 0.7). The genes with the strongest positive expression correlations with *PD-L1* included *CD84*, *PDCD1LG2*, and *JAK2* (0.6 < *r* < 0.7). *CD84* (CD84 molecule) encodes a membrane glycoprotein belonging to the signaling lymphocyte activation molecule (SLAM) family. Prior studies have shown that CD84 can upregulate PD-L1 expression in cancer (Lewinsky et al., 2018). Like *PD-L1*, *PDCD1LG2* (programmed cell death 1 ligand 2) encodes a ligand of PD-1 (programmed cell death 1). It is reasonable that this gene has a significant positive expression correlation with *PD-L1*. *JAK2* (Janus kinase 2) encodes a non-receptor tyrosine kinase having a pivotal role in cytokine and growth factor signaling. This gene has been shown to be co-upregulated with *PD-L1* in cancer (Ikeda et al., 2016). On the other hand, although there were significant negative correlations between numerous genes’ expression and CD8^+^ T cell enrichment or cytolytic activity in HCC, these correlations were not strong (FDR <0.05; *r* > −0.4).

### Identification of proteins whose expression correlates with antitumor immune responses in hepatocellular carcinoma

**FIGURE 3:**
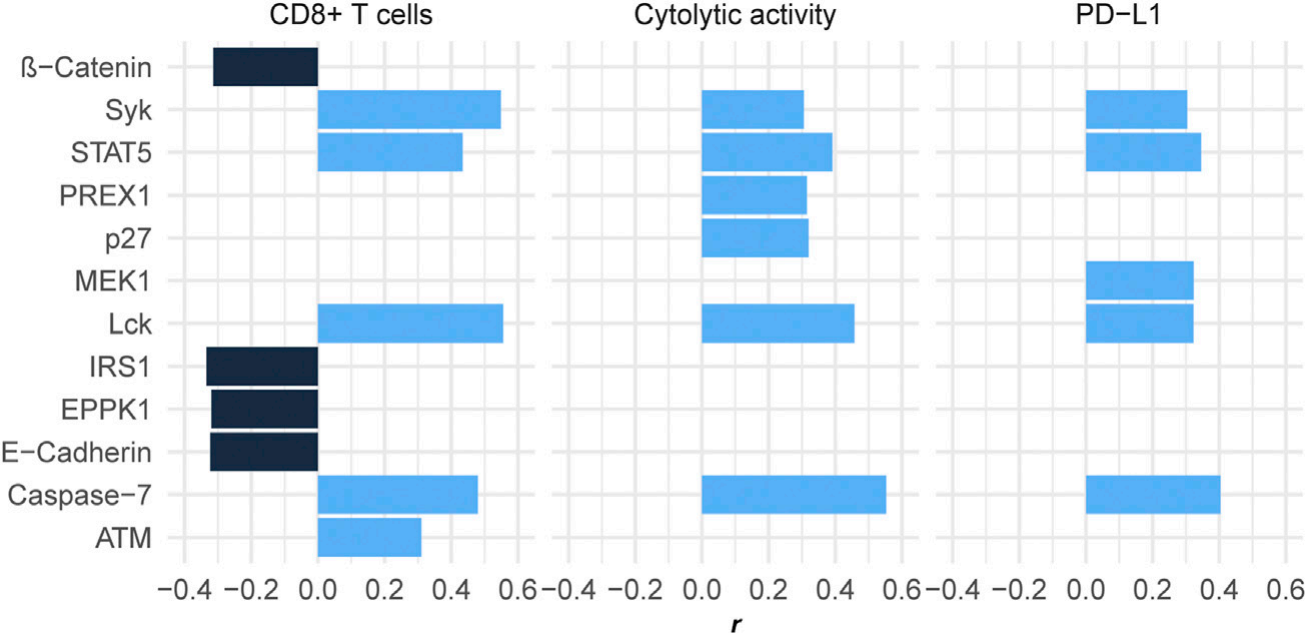
Proteins having significant expression correlations with CD8^+^ T cell enrichment, cytolytic activity, or *PD-L1* expression in HCC (|*r*| > 0.3). The Pearson correlation coefficients (*r*) are shown.

We found five proteins showing significant positive expression correlations with the enrichment levels of CD8^+^ T cells in HCC (*r* > 0.3) (Figure 3). These proteins included ATM, STAT5, Caspase-7, Syk, and Lck. There were six proteins whose expression correlated significantly and positively with cytolytic activity (*r* > 0.3) (Figure 3), including Syk, PREX1, p27, STAT5, Lck, and Caspase-7. Caspase-7 is involved in the regulation of apoptosis (Lamkanfi and Kanneganti, 2010) that is positively associated with antitumor immune responses (Jiang et al., 2019). PREX1 is a guanine nucleotide exchange factor for the RHO family of small GTP-binding proteins (RACs), which are involved in the modulation of antitumor immune responses (Li et al., 2020b). Lck is a key signaling molecule regulating T cell development, which is able to promote antitumor immune responses (Duan et al., 2021). p27 is a member of the family of KIP1 inhibitors, which plays a role in tumor suppression by inhibiting cell cycle and promoting apoptosis (Lloyd et al., 1999). Again, it is justified that p27 has a significant positive correlation with antitumor immune responses for its role in inciting apoptosis. Like p27, ATM also play a role in promoting apoptosis and suppressing tumor development (Pusapati et al., 2006). Syk is as well a tumor suppressor and play an important role in tumor immune regulation (Krisenko and Geahlen, 2015). STAT5 has also been shown to promote antitumor immunity (Ding et al., 2020).

We found four proteins whose expression had significant negative correlations with CD8^+^ T cell enrichment and/or cytolytic activity in HCC (*r* < −0.3), including IRS1, E-Cadherin, EPPK1, and β-Catenin (Figure 3). It indicates that the upregulation of these protein may inhibit antitumor immune responses in HCC. In fact, previous studies have demonstrated their oncogenic and antitumor immunosuppressive roles (Bouameur et al., 2014; Jinesh et al., 2017; Wang et al., 2020; Hong et al., 2021).

In addition, we found five proteins showing significant positive expression correlations with *PD-L1* in HCC, including Syk, Lck, MEK1, STAT5, and Caspase-7 (*r* > 0.3) (Figure 3). Interestingly, four of the five proteins (Syk, Lck, STAT5, and Caspase-7) also had significant positive expression correlations with CD8^+^ T cell enrichment and cytolytic activity in HCC. Because both PD-L1 expression (Taube et al., 2014) and TIL abundance (Haanen, 2017) are positive predictors for the response to immune checkpoint inhibitors (ICIs), the upregulation of Syk, Lck, STAT5, and Caspase-7 could indicate a better immunotherapeutic response in HCC patients.

### Identification of miRNAs whose expression correlates with antitumor immune responses in hepatocellular carcinoma

**FIGURE 4:**
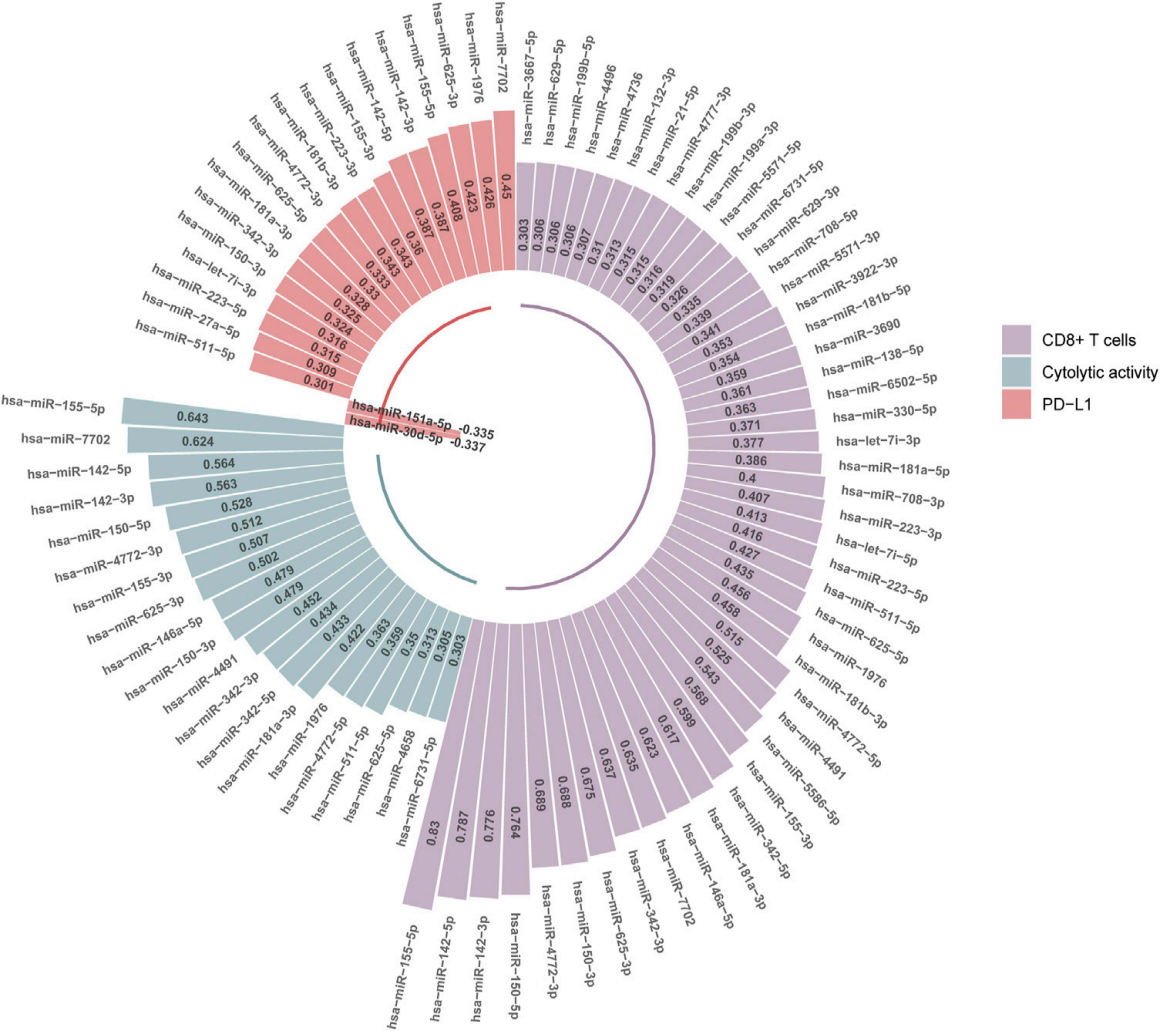
MicroRNAs (miRNAs) having significant expression correlations with CD8^+^ T cell enrichment, cytolytic activity, or *PD-L1* expression in HCC (|*r*| > 0.3). The Pearson correlation coefficients (*r*) are shown.

We found 47 and 20 miRNAs whose expression levels had significant positive correlations with CD8^+^ T cell enrichment and cytolytic activity in HCC, respectively (*r* > 0.3) (Figure 4). Among them, 19 miRNAs displayed significant positive expression correlations with both CD8^+^ T cell enrichment and cytolytic activity in HCC, including hsa-miR-6731-5p, 625-5p, 511-5p, 4772-5p, 1976, 181a-3p, 342-5p, 342-3p, 4491, 150-3p, 146a-5p, 625-3p, 155-3p, 4772-3p, 150-5p, 142-3p, 142-5p, 7702, 155-5p. Of note, hsa-miR-155-5p had the highest positive expression correlation with CD8^+^ T cell enrichment (*r* = 0.83) and with cytolytic activity (*r* = 0.64). This miRNA gene has been shown to be capable of boosting antitumor immune responses (Michaille et al., 2019), thus consistent with our results. The hsa-miR-142 genes hsa-miR-142-5p and hsa-miR-142-3p had the second and third highest positive expression correlations with CD8^+^ T cell enrichment in HCC (*r* = 0.79 and 0.78, respectively), and the third and fourth highest positive expression correlations with cytolytic activity (both *r* = 0.56). Previous studies have shown that hsa-miR-142 has a role in promoting antitumor immune responses (Jia et al., 2017; Berrien-Elliott et al., 2019), in line with our results. The miRNA gene hsa-miR-150-5p displayed significant positive expression correlations with both CD8^+^ T cell enrichment and cytolytic activity in HCC (*r* > 0.5). Again, this is consistent with previous reports showing that hsa-miR-150 has a strong association with antitumor immune responses (Enerly et al., 2011).

We found 20 miRNAs having significant expression correlations with *PD-L1* in HCC (|*r*| > 0.3), with 18 positive and 2 negative correlations, respectively (Figure 4). Interestingly, 13 of the 18 miRNAs were also significantly and positively correlated with CD8^+^ T cell enrichment and cytolytic activity in HCC. These miRNAs included hsa-miR-511-5p, 150-3p, 342-3p, 181a-3p, 625-5p, 4772-3p, 155-3p, 142-5p, 142-3p, 155-5p, 625-3p, 1976, 7702. Again, these miRNAs could be potentially useful biomarkers for immunotherapeutic responses in HCC patients.

### Identification of LncRNAs whose expression levels are associated with antitumor immune responses in hepatocellular carcinoma

**FIGURE 5:**
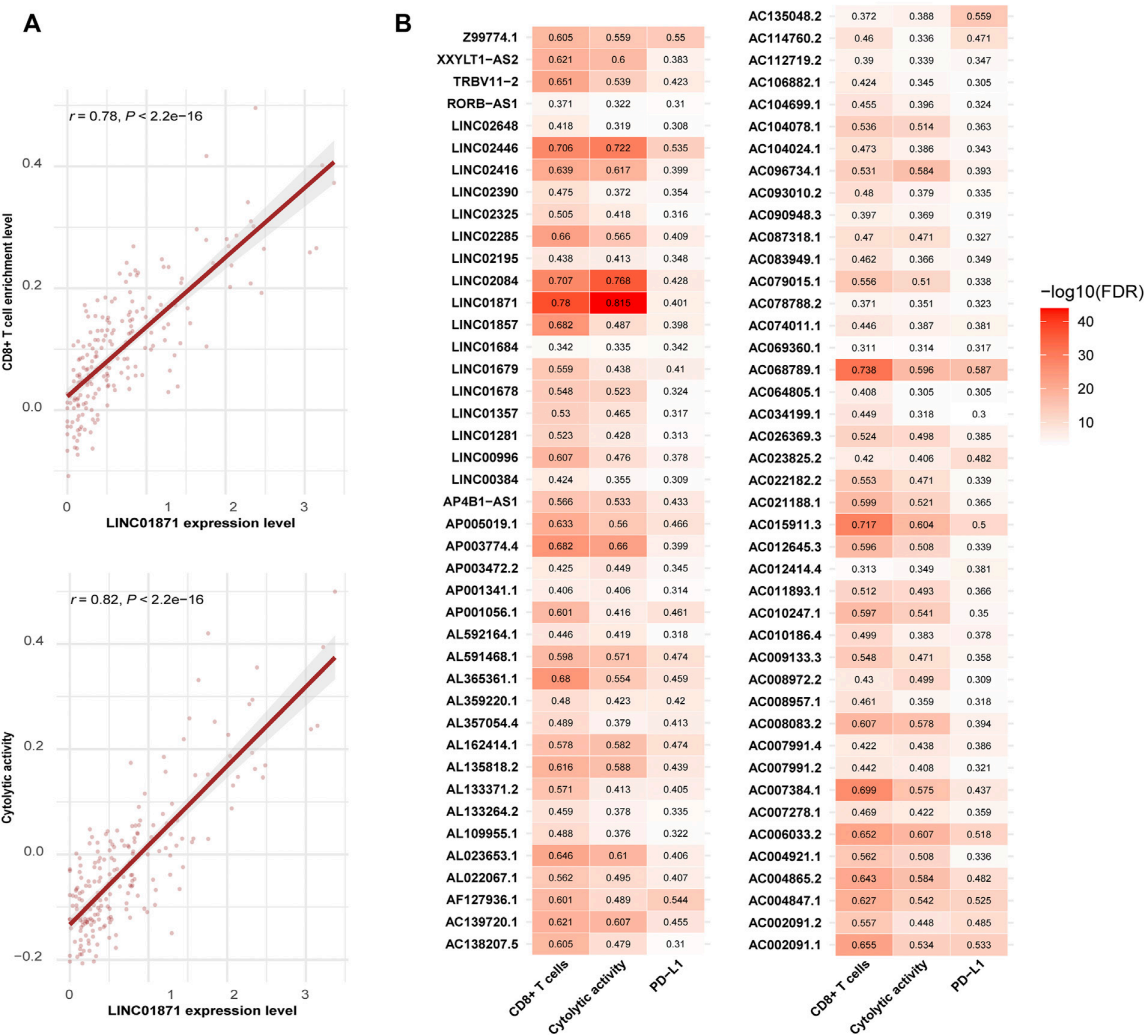
Long non-coding RNAs (lncRNAs) having significant expression correlations with CD8^+^ T cell enrichment, cytolytic activity, or *PD-L1* expression in HCC (|*r*| > 0.3). **(A)** LINC01871 showing the strongest expression correlations with both CD8^+^ T cell enrichment and cytolytic activity in HCC. The Pearson correlation coefficients (*r*) and *p*-values are shown. **(B)** Heatmap showing 85 lncRNAs with significant positive expression correlations with CD8^+^ T cell enrichment, cytolytic activity, and *PD-L1* expression in HCC (*r* > 0.3).

LncRNAs have been shown to be important in immune regulation and antitumor immunity (Heward and Lindsay, 2014; Geng and Tan, 2016; Denaro et al., 2019). We identified 209 and 19 lncRNAs showing significant positive and negative expression correlations with the enrichment of CD8^+^ T cells in HCC, respectively (|*r*| > 0.3) (Supplementary Table S2). Among them, LINC01871 had the strongest expression correlation with CD8^+^ T cell enrichment (*r* = 0.78) and also the strongest expression correlation with cytolytic activity in HCC (*r* = 0.82) (Figure 5A). Further, we found 148 and 2 lncRNAs having significant positive and negative expression correlations with cytolytic activity in HCC, respectively (|*r*| > 0.3) (Supplementary Table S2). Among them, 131 lncRNAs also had significant positive expression correlations with the enrichment of CD8^+^ T cells in HCC, and 1 lncRNA (UGDH-AS1) showed a significant negative expression correlation with CD8^+^ T cell enrichment. Moreover, we found 197 and 12 lncRNAs whose expression was positively and negatively correlated with *PD-L1* expression in HCC, respectively (|*r*| > 0.3) (Supplementary Table S2). 85 of the 197 lncRNAs also had significant positive expression correlations with CD8^+^ T cell enrichment and cytolytic activity in HCC (Figure 5B). Again, it indicates that these lncRNAs could be potentially useful biomarkers for immunotherapeutic responses in HCC patients.

### Identification of cancer-associated pathways correlated with antitumor immune responses in hepatocellular carcinoma

**FIGURE 6:**
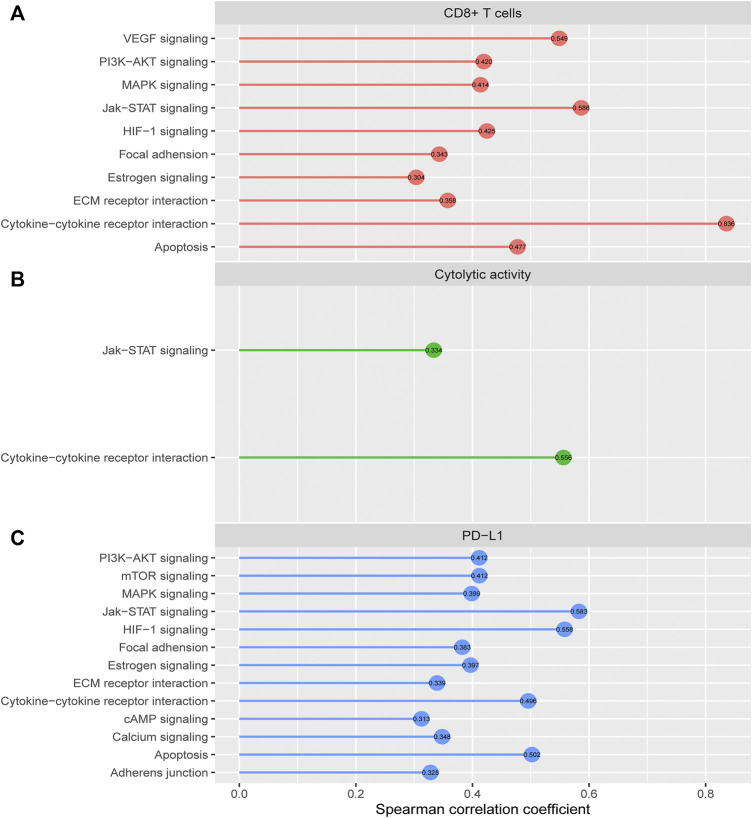
Cancer-associated pathways whose enrichment is significantly correlated with CD8^+^ T cell enrichment, cytolytic activity, or *PD-L1* expression in HCC. The cancer-associated pathways having significant positive correlations of their enrichment with CD8^+^ T cell enrichment **(A)**, cytolytic activity **(B)**, and *PD-L1* expression **(C)** in HCC (*r* > 0.3).

We identified 10 cancer-associated pathways whose enrichment was positively correlated with CD8^+^ T cell enrichment in HCC (Spearman correlation coefficient (*ρ*) > 0.3) (Figure 6A). These pathways included apoptosis, cytokine-cytokine receptor interaction, Jak-STAT signaling, MAPK signaling, VEGF signaling, PI3K-AKT signaling, HIF-1 signaling, ECM receptor interaction, focal adhesion, and estrogen signaling. In these pathways, apoptosis, cytokine-cytokine receptor interaction, and Jak-STAT signaling are immune relevant. The HIF-1 signaling pathway is upregulated in diverse cancers and may activate tumor‐associated immune signatures (Balamurugan, 2016). Moreover, a previous study (Jiang et al., 2019) showed that tumor glycolysis correlated positively with antitumor immune signatures in various cancers, supporting that the glycolysis-stimulating HIF-1 signaling correlated positively with antitumor immune responses in HCC. The positive correlation between the other pathways (focal adhesion, MAPK signaling, VEGF signaling, PI3K-AKT signaling, ECM receptor interaction, and estrogen signaling) and antitumor immune responses has been revealed in previous studies (Jiang et al., 2018; Lyu et al., 2019). In addition, we found 2 cancer-associated pathways whose enrichment was positively correlated with cytolytic activity in HCC (*ρ* > 0.3), including cytokine-cytokine receptor interaction and Jak-STAT signaling (Figure 6B). There were 13 pathways whose enrichment was significantly and positively correlated with *PD-L1* expression (*ρ* > 0.3) (Figure 6C). These pathways included Jak-STAT signaling, HIF-1 signaling, apoptosis, cytokine-cytokine receptor interaction, mTOR signaling, PI3K-AKT signaling, MAPK signaling, estrogen signaling, focal adhesion, calcium signaling, ECM receptor interaction, adherens junction, and cAMP signaling. Interestingly, many of these pathways also had significant positive correlations with CD8^+^ T cell enrichment, such as apoptosis, cytokine-cytokine receptor interaction, Jak-STAT signaling, MAPK signaling, PI3K-AKT signaling, HIF-1 signaling, ECM receptor interaction, focal adhesion, and estrogen signaling. It indicates that these pathways’ upregulation could be associated with a better immunotherapeutic response in HCC patients.

### Associations of TMB and TALs with antitumor immune responses in hepatocellular carcinoma

**FIGURE 7:**
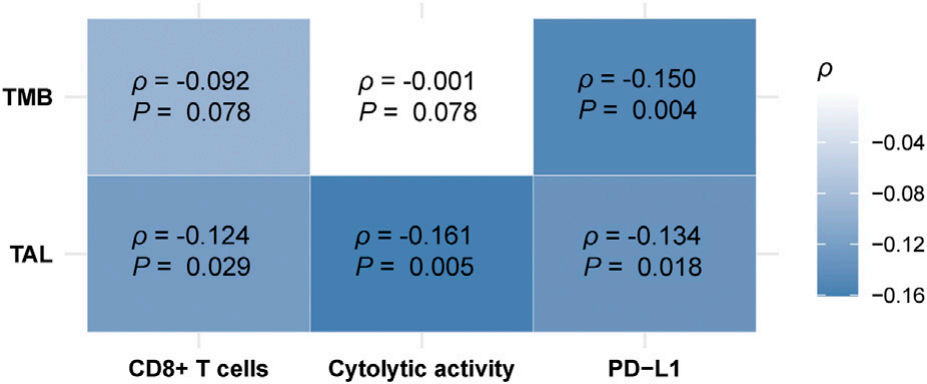
Correlations of tumor mutation burden (TMB) and tumor aneuploidy levels (TALs) with CD8^+^ T cell enrichment, cytolytic activity, and *PD-L1* expression in HCC. Heatmap showing that TALs have significant negative correlations with CD8^+^ T cell enrichment, cytolytic activity, and *PD-L1* expression, and TALs have a significant negative correlation with *PD-L1* expression in HCC. The Spearman correlation coefficients (*p*) and *p*-values are shown.

TMB has been shown to have a positive association with antitumor immunity and immunotherapy response (Samstein et al., 2019). In contrast, TALs often have an inverse correlation with them (Davoli et al., 2017). We found that TALs had significant negative correlations with both CD8^+^ T cell enrichment and cytolytic activity in HCC (Spearman correlation, *p* < 0.03) (Figure 7), supporting previous findings (Davoli et al., 2017). However, we did not observe significant positive correlation between TMB and CD8^+^ T cell enrichment or cytolytic activity in HCC (*p* > 0.05) (Figure 7). It supports previous findings that the association between TMB and antitumor immunity is cancer type dependent (Wang and Li, 2019). Interestingly, we found that both TMB and TALs correlated negatively with *PD-L1* expression in HCC (Figure 7). This analysis indicates that TMB could not be a good predictor for the response to anti-PD-1/PD-L1 immunotherapies. This indication conforms to a previous report that high TMB fails to predict the response to ICIs across all cancer types (McGrail et al., 2021).

## Discussion

In this study, we identified numerous genes, miRNAs, lncRNAs, proteins, and pathways having mutation, expression, or enrichment correlations with antitumor immunity in HCC. We found several oncogenes or tumor suppressor genes whose mutations were associated with reduced antitumor immunity in HCC, including *TTN*, *TP53*, *CTNNB1*, *RB1*, and *ZFHX4*. Among them, *TP53* and *RB1* are famed tumor suppressor genes (Sherr and McCormick, 2002). Our analysis suggests that the loss of *TP53* or *RB1* function may result in antitumor immunosuppression in HCC, consistent with the findings shown in other cancers (Jiang et al., 2018; Xiao et al., 2018; Lyu et al., 2019). *CTNNB1* is an oncogene whose hyperactivation is associated tumor progression in HCC (Rebouissou et al., 2016). Our analysis suggests that *CTNNB1* mutations may inhibit antitumor immune responses and that the upregulation of the Wnt/β-catenin pathway can compromise antitumor immunity. To our best knowledge, for the first time, we uncovered that *ZFHX4* mutations are associated with an immunosuppressive TME in HCC. This finding is noteworthy since *ZFHX4* is frequently mutated in multiple cancer types (Sun et al., 2018) and the association between its mutations and antitumor immunity in other cancer types is worthy of investigation.

We found four proteins (Syk, Lck, STAT5, and Caspase-7) whose expression correlated positively with CD8^+^ T cell enrichment, cytolytic activity, and PD-L1 expression in HCC. It suggests that these proteins’ expression could be useful biomarkers for the response to ICIs since both PD-L1 expression (Taube et al., 2014) and TIL abundance (Haanen, 2017) are positively associated with the response to ICIs. Similiarly, we identified other types of biomarkers potentially useful for predicting the response to ICIs, including miRNAs (hsa-miR-511-5p, 150-3p, 342-3p, 181a-3p, 625-5p, 4772-3p, 155-3p, 142-5p, 142-3p, 155-5p, 625-3p, 1976, 7702), many lncRNAs, as well as pathways (apoptosis, cytokine-cytokine receptor interaction, Jak-STAT signaling, MAPK signaling, PI3K-AKT signaling, HIF-1 signaling, ECM receptor interaction, focal adhesion, and estrogen signaling).

PD-L1 is expressed on the surface of immune and cancer cells and inhibits proliferation and function of T cells by binding to its receptor PD-1 (Chen et al., 2019). PD-L1 expression is deemed to be a predictive biomarker for immunotherapeutic responses in cancer (Davis and Patel, 2019). Evidences have confirmed that some miRNAs can directly or indirectly regulate the expression of PD-L1. For example, miR-200 and miR-34a directly inhibit the PD-L1 expression in non-small cell lung cancer (NSCLC) (Chen et al., 2014; Cortez et al., 2016). In contrast, miR-20b, miR-21, and miR-130b have been associated with PTEN repression in colorectal cancer, which in turn promotes PD-L1 upregulation (Zhu et al., 2014). Additionally, there are some evidences showing that miRNA can regulate CTLA-4 and PD-1 to influence the antitumor immune responses (Romano and Kwong, 2018). Furthermore, some studies have shown that miRNAs are useful in predicting the response to ICIs. For example, Rajakumar et al. defined a 5-miRNA risk score which outperformed the tissue-based PD-L1 staining in predicting overall survival following immunotherapy in advanced NSCLC (Rajakumar et al., 2022). Fan et al. found that in NSCLC treated with anti-PD-1 antibody, miR-93, miR-138-5p, miR-200, miR-27a, miR-424, miR-34a, miR-28, miR-106b, miR-193a-3p, and miR-181a were significantly upregulated in responders *versus* non-responders (Fan et al., 2020). It indicates that the alterations in circulating miRNAs are associated with the response to ICIs. Although the application of miRNAs and lncRNAs in predicting the response to ICIs in HCC remains unclear, evidences have demonstrated that various miRNAs and lncRNAs are associated with the invasion, metastasis and chemosensitivity of HCC (Gupta et al., 2020). Meanwhile, the important role of miRNAs and lncRNAs in immune regulation and antitumor immunity indicates their potential in predicting the response to ICIs in HCC.

Thus, considering that current immunotherapies for HCC have achieved some but limited successes (Liu et al., 2021), these candidate biomarkers may provide important clinical values. However, further experimental and clinical investigations are must to validate the current findings.

## Conclusion

This analysis uncovered various types of molecular features (genes, miRNAs, lncRNAs, proteins, and pathways) whose expression or mutations had significant correlations with HCC immunity. These molecular features could be predictive biomarkers for immunotherapeutic responses in HCC patients. They could also be potential intervention targets for boosting antitumor immunity and immunotherapeutic responses in HCC. Our data provide new insights into the tumor biology of HCC and potential clinical implications for the management of this disease.

## Data Availability

The original contributions presented in the study are included in the article/Supplementary Material, further inquiries can be directed to the corresponding author.
